# Effects of media exposure on PTSD symptoms in college students during the COVID-19 outbreak

**DOI:** 10.3389/fpubh.2023.1050759

**Published:** 2023-05-09

**Authors:** Xiao-Li Zhu, Zhu Wen, Wen-Bo Yu

**Affiliations:** ^1^Department of Students Affair, Nanjing Institute of Technology, Nanjing, Jiangsu, China; ^2^Department of Psychology, Children’s Hospital of Fudan University, Shanghai, China; ^3^Shanghai Mental Health Center, Shanghai Jiao Tong University School of Medicine, Shanghai, China; ^4^Department of Neurology and National Research Center for Aging and Medicine & National Center for Neurological Disorders, State Key Laboratory of Medical Neurobiology, Huashan Hospital, Fudan University, Shanghai, China

**Keywords:** media content, mental health, COVID-19, post-traumatic stress disorder, college students, media exposure

## Abstract

**Objective:**

We aimed to investigate the influence of media on college students’ mental health during the COVID-19 pandemic.

**Methods:**

After the COVID-19 outbreak, we used cross-sectional surveys through online questionnaires to investigate the mental health of college students in lockdown at home. We identified the influencing factors of PTSD symptoms using the Chi-Square test and ordinal logistic regression analysis.

**Results:**

In 10,989 valid questionnaires, 9,906 college students with no PTSD symptoms, 947 college students with subclinical PTSD symptoms (1–3 items), and 136 college students with four or more PTSD symptoms were screened out. The results showed that media content impacted the mental health of college students in lockdown at home. Positive media content was negatively correlated with PTSD symptoms among college students. PTSD symptoms were not associated with sources of information. Moreover, College students with PTSD symptoms would reduce their willingness to learn and could not complete online learning efficiently.

**Conclusion:**

PTSD symptoms are related to media exposure and excessive information involvement of COVID-19 in college students, which influences the willingness to attend online classes.

## Introduction

“Coronavirus Disease” was first diagnosed in December 2019 and officially named “COVID-19” on February 11, 2020 (1), which still has a massive impact on global public health and healthcare to date (2, 3). When COVID-19 broke out in China, the country’s public health policy adopted locking down cities to stop the spread of the virus, and the policy had a pronounced effect on blocking the spread of the virus in the population. Until now, China has been the only country that insists on this policy. However, the sudden and continuous lockdown not only causes economic losses and inconvenience in life, work, and transportation but also affects the emotional state of individuals living in these areas, thus having a clear impact on mental health (4, 5). In March 2022, China again implemented long-term and large-scale closure measures in cities, including Shanghai, indicating that centralized isolation or quarantine at home is still the main China epidemic prevention policy. Therefore, the impact of this epidemic prevention strategy on psychiatric disorders still deserves our continued attention.

Among these most common psychiatric disorders, Post-traumatic stress disorder (PTSD) is one of the more serious mental illnesses. The prevalence of PTSD following the COVID-19 outbreak has been widely documented. The incidence of PTSD was 10.4% among adolescents, 16.3% among master’s and doctoral students, 3.16% among children, and 3.53% among parents (6). After a month of lockdown, 19.5% of French university students have PTSD (7). In a meta-analysis of nearly 100,000 healthcare workers in 21 countries, the incidence of PTSD was 21.5% (8). The researchers screened 421 Chinese healthcare workers for PTSD after the outbreak of COVID-19. They found that 13.2% had PTSD symptoms, and only in low-risk areas, non-medical staff has risk factors for PTSD symptoms (9).

College students are a relatively particular group affected by the lockdown measures. Since the lockdown Policy, most students began a long period of school suspension, quarantined at home, and online courses. Nevertheless, most college students leave their hometown [91.1% (10009/10989) in this study] to study in other cities or provinces (10). Their peer social networking or interpersonal circle depends more on the school classmate group. The impact on academics and social interaction is more prominent with lockdown measures and restricted travel. Also, the primary age group of college students is 17–23. This age group also has various emotional disorders and mental health problems, such as bipolar disorder, anxiety, depression, insomnia, and Post-traumatic stress disorder (11, 12). When COVID-19 first became popular, an online questionnaire study found that among 5,982 medical students in school, moderate to severe depression occurred. The symptom rate was 35.2%; mild-to-severe anxiety was 22.8%; students with poor social support were also found to be more severely ill (13). During the mass lockdown, most staff was quarantined at home. It is difficult for colleges to carry out mental health censuses, let alone related interventions. Therefore, online questionnaires for college students’ mental health screening are a few practical approaches. Based on the above reasons, this study focuses on college students. The online questionnaires surveyed the mental health of more than 10,000 college students during the lockdown period.

According to the DSM-5, PTSD is a mental and behavioral disorder that develops in individuals following one or more traumatic events. Specific symptoms may include avoidance of reactions to triggering events, changes in mood and cognition, perception of imminent threat, disturbed sleep, and hypervigilance (14). PTSD is a long-term mental disorder of delayed onset. Symptoms of PTSD typically begin within 6 months after trauma (3.8%) and persist through at least 1 month; some can reach 12 months, with a prevalence of ~4.7% and a lifetime risk of 8.3% (15). In modern society, human beings are often faced with public events or natural disasters, and the relevant media coverage is generally sufficient and continuous, but individuals may often have excessive media usage and inappropriate attention to harmful media content. For this reason, the odds of PTSD, trauma, and stress-related illnesses significantly increase after public events or natural disasters (16–19). Research by Rebecca R. Thompson et al. found that after major violent events (massacres, bombings), people exposed to the media will feel increased stress and distress, and media exposure to related events within a few months will increase their susceptibility to PTSD. Therefore, researchers point out that trauma-related media exposure can lead to a long-term cycle of high stress and media use (20). Brian J. Hall et al. proposed that media exposure after major natural disasters can indirectly impact PTSD. His research found that after typhoons, the prevalence of PTSD was 5.1%. Media coverage of residents’ emotional reactions and information about drowning victims raised the risk of PTSD, while reports of heroic deeds and the storm were associated with lower odds of PTSD (21). Therefore, media use and certain content may impact individuals’ physical and mental health. The consequences of PTSD caused by media exposure are severe over time, comprehensively affecting peoples’ learning and social functions. Currently, there is less research on the impact of media on PTSD under China’s lockdown policy (22–24). The former research shows that for college students, the incidence of PTSD in previous studies was 6–17%, and the main risk factors were: female and socioeconomic status (25). During the lockdown caused by COVID-19, most people, including college and middle school students, are affected by media information and experience various psychological reactions, such as insomnia, anxiety, panic, confusion, irritability, and even PTSD (4, 6, 23, 26, 27). Gao et al. surveyed the degree of public media exposure and mental health status during the COVID-19 incident among 4,827 adults. They found that compared with media exposure within 1 h, media exposure for more than 3 h can lead to a 1.30-fold and 1.23-fold increase in the risk of moderate anxiety and depression, respectively (22).

During the COVID-19 pandemic, media is the primary way for people to obtain relevant information. Everyone has to pay more attention to news reports and track various media information, which are the issues that everyone has to face. Because college students are familiar with various electronic information sources, they use social media most actively. So they are easy to contact and be influenced by external information through various channels, including traditional media (newspapers, TV, mobile news), social media, and direct human-to-human conversations (28–34).

On traditional and social media, much news about COVID-19 emerges 24 h a day. Some studies have shown that continuous media exposure after public events or natural disasters is related to the psychological problems of exposed people (16, 17, 35–43). Furthermore, this tendency to search for information will, in turn, affect media exposure. Nowadays, social media platforms generally use recommendation systems to push content that viewers care about, making it easier for users to see the content they are concerned about. This reality has led to the mutual influence of media and mental health. While evaluating the impact of epidemic-related media reports and quarantines on college students’ mental health, we found that “media content” relates to PTSD. What are the main influencing factors? There are fewer related studies and even less research on “media content” with a separate study. Therefore, we focused our research on the impact of media content on PTSD symptoms in college students in the early stages of the COVID-19 outbreak. We hope our research can help and guide how media content can be adapted to support mental health during the disaster better.

## Methods

### Source and study population

This research is a cross-sectional study of valid online questionnaires submitted from March 10, 2020, to April 9, 2020. The participating students are (1) registered at Nanjing Institute of Technology (2), voluntarily participating in this study and completing the online questionnaire (additional details of the questionnaire can be found in Appendix A), Students on maternity leave and long-term sick leave were excluded from the study. We sent an online questionnaire to 25,609 students. About 11,011 questionnaires were collected, with a response rate of 42.9%. After excluding students who did not provide valid information, 10,989 valid questionnaires were included in the analysis. Among them, 7,264 were male (66.1%), and 3,725 were female (33.90%).

### Measurement

The Short Screening Scale was used as a self-report measure for PTSD symptoms and was revised in 1998 by Naomi Breslau et al. The Short Screening Scale mainly involves the screening self-rating scale for Post-traumatic stress disorder, including seven main symptoms, five symptom items are related to avoidance and numbness, and two are related to hyperarousal. A score of 4 or higher was judged as screening positive. In PTSD-positive cases aged 18–45 years old, the sensitivity was 80%, the specificity was 97%, the positive predictive value was 71%, and the negative predictive value was 98% (44). The scale is an effective and convenient clinical screening tool for PTSD symptoms (45–47). Based on this score, we divided all people into three groups, group 1 with no PTSD symptoms, group 2 with one to three PTSD symptoms, and group 3 with four or more PTSD symptoms.

According to the residence region of the participating students, the participants are divided into two categories: those in Hubei Province and those not in Hubei Province. Sociodemographic data were collected, including age, gender (male/female), and class presidency or class cabinet. The information related to COVID-19 includes knowledge about COVID-19, the quarantine period, the number of masks used during the quarantine period, the attitude toward online courses, and the willingness to return to school. Media exposure-related information includes the time point of beginning to pay attention to COVID-19, the main ways to know the latest developments of the epidemic, what information you most want to know in your home quarantine, and reasons for optimism.

### Data analysis

First, a descriptive analysis was performed to understand the characteristics of the sample and determine the prevalence of PTSD symptoms. Second, the proportion of students in different groups was calculated by PTSD score. Count data is expressed in quantity (percentage). Third, chi-square analysis was used to screen out the factors related to PTSD groups preliminarily. Forth, an ordinal logistic regression model was established to test the influence of geographical, sociodemographic, media content, and other factors on the PTSD groups. Two different models were established by ordinal logistic regression analysis. Model 1 was a model that included only seven reasons associated with optimism. Model 2 not only includes the seven reasons of Model 1 but also includes gender, the time point of beginning to pay attention to COVID-19, and the region of residence (Hubei province). These three factors have statistical differences in the chi-square test. Last, ordinal logistic regression was used to analyze the PTSD group relation to attitude toward online courses and willingness to return to school. All analyses were performed using SPSS software (version 22.0, IBM, Armonk, NY, United States). The statistical significance threshold *p* was 0.05. The risk coefficient (RO), associated two-tailed *p*-value (*p*), and 95% confidence interval (CI) were parameters in the regression model.

## Results

### Epidemiological characteristics

Table 1 summarizes the general demographic characteristics of all participants and results in the survey, presented by the number and percentage (n/%) of each group. In a total 10,989 college students, group 1 (without PTSD symptoms) was 9,906 (90.1%); group 2 (with 1–3 PTSD symptoms) was 947 (8.3%); group 3 (with 4 or 3 PTSD symptoms) was 136 cases (1.2%).

**Table 1:** Characteristics of cases

	**Study Groups**				**Statistical Analysis**	
	**group 1:No PTSD Symptoms(n=9906[90.1%])**	**group2:1-3 PTSD Symptoms(n=947 [8.6%])**	**group3:≥4 PTSD Symptoms(n=136 [1.2%])**	**All(n=10989)**	**χ** ^**2**^	***P***
**Age**						
**<18y**	63(0.64)	7(0.74)	0(0.00)	70(0.64)	5.414	0.492
**18**	1070(10.80)	102(10.77)	8(5.88)	1180(10.74)		
**19**	2402(24.25)	223(23.55)	39(28.68)	2664(24.24)		
**>=20**	6371(64.31)	615(64.94)	89(65.44)	7075(64.38)		
**Gender**						
**male**	6634(66.97)	555(58.61)	75(55.15)	7264(66.10)	34.358	0.000^***^
**female**	3272(33.03)	392(41.39)	61(44.85)	3725(33.90)		
**Region of residence**						
**None Hubei province**	9836(99.29)	930(98.20)	136(100.00)	10902(99.21)	14.138	0.001^**^
**Hubei province**	70(0.71)	17(1.80)	0(0.00)	87(0.79)		
**Class president or class cabinet**						
**Class president**	586(5.92)	62(6.55)	6(4.41)	654(5.95)	8.128	0.229
**Student Union leader**	930(9.39)	90(9.50)	17(12.50)	1037(9.44)		
**Other committee leader**	2323(23.45)	249(26.29)	36(26.47)	2608(23.73)		
**None**	6067(61.25)	546(57.66)	77(56.62)	6690(60.88)		
**Knowledge about COVID-19**						
**A lot**	4212(42.52)	398(42.03)	47(34.56)	4657(42.38)	4.033	0.402
**A little**	5659(57.13)	545(57.55)	88(64.71)	6292(57.26)		
**None**	35(0.35)	4(0.42)	1(0.74)	40(0.36)		
**Quarantine period**						
**1-3d**	1663(16.79)	155(16.37)	27(19.85)	1845(16.79)	7.202	0.515
**4-7d**	1431(14.45)	140(14.78)	16(11.76)	1587(14.44)		
**8-15d**	1714(17.30)	172(18.16)	16(11.76)	1902(17.31)		
**16-30d**	1870(18.88)	168(17.74)	33(24.26)	2071(18.85)		
**>30d**	3228(32.59)	312(32.95)	44(32.35)	3584(32.61)		
**During the quarantine, number of masks used**						
**10 or less**	1140(11.51)	133(14.04)	22(16.18)	1295(11.78)	8.2	0.085
**10-20**	3290(33.21)	303(32.00)	40(29.41)	3633(33.06)		
**20 or more**	5476(55.28)	511(53.96)	74(54.41)	6061(55.16)		
**Attitude toward online courses**						
**Like**	4521(45.64)	339(35.80)	40(29.41)	4900(44.59)	80.441	0.000^***^
**Just to listen**	3759(37.95)	374(39.49)	52(38.24)	4185(38.08)		
**A task must be completed**	629(6.35)	98(10.35)	19(13.97)	746(6.79)		
**Do not like**	997(10.06)	136(14.36)	25(18.38)	1158(10.54)		
**Willingness to return to school**						
**Be willing to**	5697(57.51)	556(58.71)	76(55.88)	6329(57.59)	5.946	0.203
**whatever**	3837(38.73)	351(37.06)	50(36.76)	4238(38.57)		
**Do not want to return to school**	372(3.76)	40(4.22)	10(7.35)	422(3.84)		

χ2 test for categorical variables to assess differences across PTSD groups. Abbreviations: PTSD, posttraumatic stress disorder.^**^P<0.01,^***^p < 0.001.

There was less proportion of age 18 and under 18 in group 3 (5.88%) than in groups 1 (11.44%) and 2 (11.51%). Conversely, the age group of 19 and over 20 years old in group 3 (94.12%) is lower than in groups 1 (88.56%) and 2 (88.49%). However, it has no statistical significance.

We found that gender was significantly associated with PTSD symptoms (*χ*^2^ = 34.358, *p* = 0.000), and females were more likely to have more PTSD symptoms. There was 41.39% in group 2 and 44.85% in group 3, higher than the population participating female in the survey (33.9%).

The region and PTSD symptoms significantly correlate (*χ*^2^ = 14.138, *p* = 0.001). Hubei Province was more likely to have PTSD symptoms, and the proportion of group 2 (1.8%) was higher than that of all participants (0.79%). However, there was no Hubei Province student in group 3, which may be the limitation of voluntary participation in the online survey (see the discussion section).

### The impact of PTSD symptoms on online courses

According to the preliminary statistics results (Table 1), we found that PTSD symptoms are also significantly related to the willingness of college students to take online classes (*χ*^2^ = 80.441, *p* = 0.000). The proportions of groups 1 and 2 who want to take online classes are 45.64 and 35.8%, respectively. Group 3 (29.41%) was significantly lower than group 1 and group 2. On the other hand, group 3, who did not want to take online classes, was 18.38%, which was higher than group 1 (10.06%) and group 2 (14.36%). So, the more PTSD symptoms, the more reluctant to take online classes.

We further evaluated the effect of the PTSD group on attitudes toward online courses in the regression analysis of the PTSD group, as shown in Table 2. Compared with students who “liked online classes,” the PTSD group increased the relative risk of all negative attitudes (just to listen: OR = 1.301, 95% CI = 1.148–1.475, *p* < 0.001; a task must be completed: OR = 1.981, 95%CI = 1.647–2.382, *p* < 0.001; do not like: OR = 1.768, 95%CI = 1.5–2.085, *p* < 0.001).

**Table 2:** Association between PTSD groups and risk of attitude on online courses.

	Attitude toward online courses								
	Just to listen			A task must be completed			Do not like		
	OR	OR (95% CI)	*p*-value	OR	OR (95% CI)	*p*-value	OR	OR (95% CI)	*p*-value
PTSD group	1.301	1.148–1.475	0.000***	1.981	1.647–2.382	0.000*	1.768	1.500–2.085	0.000***

PTSD, posttraumatic stress disorder.

***p < 0.001.

The survey had no statistical difference overall in a willingness to return to school. However, the unwilling students accounted was 3.84% of the total, which was 7.35% in group 3, about double higher than group 1 (3.76%) and group 2 (4.22%). Furthermore, the students who want to go back to class are also fewer in group 3 (55.88%) than in group 2 (58.71%) and group 1 (57.51%). We also had no positive results when further regression analysis was used to explore the relationship between PTSD symptoms and willingness to return to school (Table 3).

**Table 3:** Association between PTSD groups and risk of willingness to return to school.

	Willingness to return to school					
	Whatever			Do not want to go back to school		
	OR	OR (95% CI)	*p*-value	OR	OR (95% CI)	*p*-value
PTSD group	0.956	0.855–1.069	0.432	1.239	0.966–1.590	0.091

PTSD, posttraumatic stress disorder.

The preliminary analysis did not find that PTSD symptoms were significantly correlated with age, class presidency or class cabinet, knowledge about COVID-19, quarantine period, number of masks used, and willingness to return to school (Table 1).

### PTSD symptoms and social media exposure

Table 4 focuses on the association of media exposure with PTSD symptoms. First, it was found that PTSD symptoms significantly correlated with the time point of beginning to pay attention to COVID-19 (*χ*^2^ = 21.83, *p* = 0.016). Specifically, in the early stage of the epidemic, “Wuhan Health Commission officially reported the first death case (2020.1.11)” and “Wuhan announced a “lockdown” (2020.01.23),” the proportion of students who began to pay attention to the COVID-19 at these two-time points was higher in group 3 (26.47 and 10.29%, respectively). In contrast, the students who paid attention to COVID-19 at other time points do not have this characteristic. The proportion of students who chose “other or never cared” was also higher in group 3 (5.15%).

**Table 4:** Comparisons of media exposure across PTSD groups.

	Study groups			All (*n* = 10,989)	Statistical analysis	
	group1: No PTSD Symptoms (*n* = 9,906[90.1%])	group2: 1–3 PTSD Symptoms (*n* = 947[8.6%])	group3: ≥4 PTSD Symptoms (*n* = 136[1.2%])		*χ* ^2^	*p*-value
The time point of beginning to pay attention to COVID-19						
At the end of December 2019, the Wuhan Health Commission issued a notification for the first time that 27 people were infected with viral pneumonia	2,554 (25.78)	276 (29.14)	34 (25.00)	2,864 (26.06)	21.83	0.016^*^
On January 11, 2020, the Wuhan Health Commission officially reported the first death case	2,336 (23.58)	207 (21.86)	36 (26.47)	2,579 (23.47)		
On 19 January and 20 January 2020, cases occurred in Shenzhen and Beijing	1710 (17.26)	178 (18.80)	20 (14.71)	1908 (17.36)		
On January 20, 2020, Zhong Nanshan affirmed that the new coronavirus pneumonia was human-to-human transmission and that medical staff were infected	2,139 (21.59)	179 (18.90)	25 (18.38)	2,343 (21.32)		
On January 23, 2020, Wuhan announced a “lockdown”	1,004 (10.14)	87 (9.19)	14 (10.29)	1,105 (10.06)		
Other or Never cared	163 (1.65)	20 (2.11)	7 (5.15)	190 (1.73)		
Main ways to know the latest developments of the epidemic						
Television news	8,127 (82.04)	773 (81.63)	111 (81.62)	9,011 (82.00)	3.736	0.88
Newspaper	2,335 (23.57)	226 (23.86)	37 (27.21)	2,598 (23.64)		
Weibo, WeChat and other social media	8,996 (90.81)	866 (91.45)	130 (95.59)	9,992 (90.93)		
Quora or Zhihu etc.	4,006 (40.44)	395 (41.71)	67 (49.26)	4,468 (40.66)		
Others	3,295 (33.26)	318 (33.58)	58 (42.65)	3,671 (33.41)		
What information do you most want to know in your home quarantine?						
Medical knowledge about effective prevention and protection of COVID-19	8,550 (86.31)	816 (86.17)	118 (86.76)	9,484 (86.30)	4.615	0.997
Potential risk of COVID-19 in the area of oneself, family, relatives and friends	8,404 (84.84)	810 (85.53)	118 (86.76)	9,332 (84.92)		
Efforts of doctors, nurses, NHC (National Health Commission) officials, and the police to save lives	6,729 (67.93)	647 (68.32)	106 (77.94)	7,482 (68.09)		
Operation of government to resist the COVID-19 pandemic	7,275 (73.44)	698 (73.71)	107 (78.68)	8,080 (73.53)		
Measures and experience of other countries or areas to resist the COVID-19 pandemic	5,277 (53.27)	511 (53.96)	90 (66.18)	5,878 (53.49)		
Information and statistical data on infection and spread of the COVID-19 pandemic	7,128 (71.96)	668 (70.54)	110 (80.88)	7,906 (71.94)		
Research progress of COVID-19 by professional and scientific institutions	6,368 (64.28)	602 (63.57)	101 (74.26)	7,071 (64.35)		
Analysis and interpretation of COVID-19 by experts, scholars and professionals	6,300 (63.60)	609 (64.31)	100 (73.53)	7,009 (63.78)		
Do not care about COVID-19	65 (0.66)	8 (0.84)	1 (0.74)	74 (0.67)		
Reasons for optimism						
The shock of China’s construction speed	7,830 (79.04)	702 (74.13)	105 (77.21)	8,637 (78.60)	22.825	0.029^*^
The increasing number of cured patients	7,251 (73.20)	667 (70.43)	94 (69.12)	8,012 (72.91)		
Sufficient protective appliances	6,420 (64.81)	515 (54.38)	80 (58.82)	7,015 (63.84)		
Public awareness of protection	7,717 (77.90)	641 (67.69)	91 (66.91)	8,449 (76.89)		
National unity can control the COVID-19 pandemic	8,170 (82.48)	722 (76.24)	106 (77.94)	8,998 (81.88)		
Have more time with families	4,666 (47.10)	420 (44.35)	70 (51.47)	5,156 (46.92)		
Others	293 (2.96)	29 (3.06)	11 (8.09)	333 (3.03)		

*χ*^2^ test for categorical variables to assess differences across PTSD groups. PTSD, posttraumatic stress disorder. ^*^p < 0.05.

In addition, PTSD symptoms were correlated with reasons of optimism (*χ*^2^ = 22.825, *p* = 0.029). Among the total student, the proportions of choosing “The shock of China’s construction speed,” “The increasing number of cured patients,” “Sufficient protective appliances,” “Public awareness of protection,” and “National unity can control the COVID-19 pandemic” were 78.6, 72.91, 63.84, 76.89, and 81.88%, respectively. The proportion of these options is lower in groups 2 and 3, but higher in group 1. Unexpectedly, all college students who chose “Have more time with families” as an optimistic reason were the least (46.92%). While among the three groups who chose this reason, group 3 was the highest (51.47%), higher than the other groups and all populations. In addition, group 3 (8.09%) chose the option “Other” significantly higher than the other two groups.

Regarding the main ways to know the latest news on COVID-19, there was no statistically significant relationship between different ways and PTSD symptoms. The percentage of group 3 is higher than the overall level in all news acquisition ways except television, indicating that they are unusually concerned about the media information. At the same time, we can see that there are multiple ways for college students to obtain information, and “social media” occupies the most significant proportion (90.93%), which has surpassed “television” (82%), while newspapers (23.64%) have the lowest percentage of all access to news.

Although the overall statistics did not find a significant correlation between the PTSD group and “information you most want to know in your home quarantine.” It can be found that paying attention to most of the COVID-19 information is higher in group 3. It indicated that students in this group 3 are more concerned about measures and results in various situations related to the epidemic.

The preliminary correlation analysis in Tables 1, 4 suggests that the PTSD group is related to gender, region of residence, the time point of beginning to pay attention to COVID-19, and reasons for optimism. In order to further examine the relationship between various factors and PTSD symptoms, an ordinal logistic regression model was performed based on correlation analysis. First, the correlation between the PTSD group and reasons for optimism was analyzed (Table 5, Model 1). Both “sufficient protective appliances “and “public awareness of protection “reduced the risk of PTSD symptoms in group 2 (OR = 0.769, 95%CI = 0.649–0.911, *p* = 0.002 and OR = 0.696, 95%CI = 0.583–0.832, *p* = 0.000, respectively). “public awareness of protection “also reduced the risk of PTSD symptoms in group 3 (OR = 0.556, 95%CI = 0.345–0.895, *p* = 0.016). Moreover, “Others” increased the risk in group 3 (OR = 2.53 [95%CI = 1.286–4.978], *p* = 0.007).

**Table 5:** Odds ratios of different PTSD groups by related factors compared with the No PTSD Symptom using multinomial logistic regression model.

	Group 2: 1–3 PTSD Symptoms (*n* = 947)						group 3: ≥4 PTSD Symptoms (*n* = 136)					
	Model 1^a^			Model 2^b^			Model 1^a^			Model 2^b^		
	OR	OR (95% CI)	*p*-value	OR	OR (95% CI)	*p*-value	OR	OR (95% CI)	*p*-value	OR	OR (95% CI)	*p*-value
Gender	–	–	–	1.446	1.260–1.659	0.000^***^	–	–	–	1.729	1.226–2.438	0.002^**^
The time point of beginning to pay attention to COVID-19	–	–	–	1.055	1.005–1.107	0.031^*^	–	–	–	0.976	0.865–1.100	0.688
Region of residence (Hubei province)	–	–	–	2.295	1.338–3.939	0.003^**^	–	–	–	0.000	0.000–Infinity	1.000
Reasons for optimism												
The shock of China’s speed	0.899	0.759–1.065	0.217	0.930	0.785–1.102	0.401	1.147	0.720–1.828	0.564	1.200	0.754–1.911	0.442
The number of people cured is increasing	1.074	0.914–1.262	0.388	1.048	0.892–1.232	0.567	0.937	0.614–1.430	0.764	0.920	0.604–1.401	0.698
Sufficient protective appliances	0.769	0.649–0.911	0.002^**^	0.786	0.663–0.931	0.005^**^	0.927	0.588–1.460	0.743	0.956	0.608–1.501	0.844
Public awareness of protection	0.696	0.583–0.832	0.000^***^	0.681	0.570–0.814	0.000^***^	0.556	0.345–0.895	0.016^*^	0.542	0.338–0.869	0.011^*^
National solidarity against COVID-19 pandemic	0.891	0.741–1.072	0.222	0.872	0.725–1.049	0.146	1.073	0.649–1.772	0.784	1.037	0.629–1.711	0.886
Have more time with families	1.071	0.928–1.236	0.348	1.053	0.912–1.215	0.485	1.432	0.990–2.071	0.056	1.424	0.986–2.058	0.060
Others	0.762	0.508–1.142	0.188	0.784	0.522–1.177	0.241	2.53	1.286–4.978	0.007^**^	2.691	1.365–5.308	0.004^**^

a Regression model included reasons for optimism.

b Regression model included sex, the timing to start paying attention to COVID-19, Hubei province, reasons for optimism.

^*^p < 0.05, ^**^p < 0.01, ^***^p < 0.001.

After controlling for the effects of three factors, including gender, region of residence, and “the time point of beginning to pay attention to COVID-19,” we analyzed the risk of PTSD symptoms among college students (Table 5, Model 2). The results showed that females have more odds of PTSD symptoms. In group 2, the risk of females developing PTSD symptoms was 1.446 times that of males (OR = 1.446, 95%CI = 1.260–1.659, *p* = 0.000), while the risk increased 1.729-fold in group 3 (OR = 1.729, 95%CI = 1.226–2.438, *p* = 0.002). In group 2, “the time point of beginning to pay attention to COVID-19” (OR = 1.055, 95% C = 1.005–1.107, *p* = 0.031) and region of residence (Hubei province) (OR = 2.295, 95% CI = 1.338–3.939, *p* = 0.003) were more risk to PTSD symptoms, but not significantly risk in group 3.

## Discussion

### Some factors related to PTSD symptoms in college students

In this study, an ordinal logistic regression model has been used to analyze the factors that significantly impacted the PTSD symptoms of college students during the epidemic. Among them, gender is closely related to the risk of PTSD symptoms in college students, indicating that females are more likely to have PTSD symptoms. In group 2, the risk of females developing PTSD symptoms is 1.446 times that of males, while this risk increased 1.729-fold in group 3. This result has also been confirmed in other studies (48).

In group 2, the time point of beginning to pay attention to COVID-19 and residence in Hubei province will also affect the odds of PTSD symptoms, while these factors were not added odds in group 3. This statistically significant difference may be due to the limitations of the online questionnaire survey. The sample size in Hubei is too small, leading to a selection bias. At the same time, the people with the most severe PTSD symptoms (group 3) may lack the willingness to participate online questionnaire survey due to the influence of the situations and emotions.

PTSD symptoms in college students can affect their day-to-day attitudes and perspectives. We found that PTSD symptoms are significantly correlated with the willingness of college students to take online courses, which is reflected in the lack of willingness of college students with PTSD symptoms to participate in online courses. Relative to “like “, PTSD symptoms increased the risk of “just to listen” of students by 1.301 times; the risk of “a task must be completed” increased by 1.981 times; the risk of “Do not like” increased by 1.768 times. Therefore, among college students with fewer PTSD symptoms, more students are willing to take online courses. However, there was no similar statistical difference in willingness to return to school. From groups 1 to 3, the number of students unwilling to return to school gradually increased, indicating that more severe PTSD symptoms exacerbated the students’ social inhibition and inability to complete daily learning and life tasks.

During the epidemic, Chinese schools stopped traditional offline teaching and turned to online courses. College students with PTSD symptoms lack flexibility and adaptability when faced with a crisis. They have weak adaptability to the online teaching model and a low willingness to learn online. In response to this situation, college should improve their efficiency of screening and assessment, provide more targeted support and guidance, and provide psychological counseling services. Only by paying special attention to the unique needs of college students with PTSD symptoms can they navigate the crisis successfully.

### The influence of media content on PTSD symptoms

Due to the development of modern media, the influence of media on the public has become more and more significant. Research has shown a positive correlation between media content and PTSD symptoms, and media consumption time was also a risk factor for PTSD symptoms. Whether or not previous exposure, the psychopathological state prior to disaster or trauma was also associated with PTSD symptoms (23, 49).

Some studies found an interaction between media exposure and sympathetic response. Adolescents are more likely to develop PTSD after exposure to natural calamities and artificial misfortunes in media content, which may be related to a lower threshold of sympathetic response. They are more likely to have psychopathological responses (50). Therefore, media content and duration of media exposure can more effectively influence the occurrence of PTSD symptoms in adolescents.

Mass violence and natural disasters spread through the modern media have become more common since the advent of 24-h television news. Exposure to trauma through media also has a variety of psychopathological consequences, such as anxiety, depression, and insomnia, of which PTSD is the most common (16, 21, 39, 43). The outbreak of COVID-19 has thrown people into a sea of information overload. Whether excessive exposure to information from media can damage the public’s mental health is a significant concern.

This study shows that the occurrence of PTSD symptoms is related to exposure to information. The proportion of students in group 3 who first began to pay attention to the epidemic under the impact of essential and catastrophic news (Wuhan closure, death cases) is higher than the other groups, indicating that students who started paying attention to the outbreak earlier were also associated with PTSD symptoms. Such students are more likely to develop PTSD symptoms, consistent with previous research findings (13, 27).

In addition, a significant increase in risk in the epicenter (Hubei province) suggests that other non-media information dissemination will also significantly impact PTSD symptoms. These results suggest that PTSD symptoms among college students relate to media content and media exposure. It is worth noting that it is not only related to the degree of excessive attention and the length of attention to information but also the negative impact of the news itself.

We can reasonably speculate that we did not start paying attention to the epidemic until we knew the information that “Wuhan announced a lockdown.” This sudden news will surprise students and let them not know what is happening around them. It will also quickly lead to anxiety and panic, a normal mental and psychological reaction. Therefore, college students must avoid sudden or excessive, long-term harmful information exposure to maintain mental health.

### The influence of access to media on PTSD symptoms

The advancement of technology is constantly changing how people obtain information, and the leap of technology has also broadened how people obtain information. In addition to traditional newspapers and TV, college students’ acquisition of media content extends to the Internet and social media. Through our survey, we found that the current media information channels of college students mainly tend to be Weibo, WeChat, and other social media (90.93%), followed by TV news (82.00%), and newspapers became the most minor used media (23.64%). This phenomenon is related to the reading habits changed by social media, the limited logistics of the newspaper industry during the epidemic, and the lag in newspaper news delivery.

Due to the convenience and speed of the Internet, college students have become accustomed to using Internet platforms to obtain all kinds of information they want. It is worth noting that the students with more PTSD symptoms have a higher proportion of obtaining information through social media, while there is no significant difference in the proportion of students in each group obtaining information from TV news. Because TV news reports are more generalized and moderated, it can be recommended that students prone to panic and anxiety use TV news as the primary channel to obtain information daily. It may be a better way to be adopted psychological intervention during large-scale disasters.

When faced with hot issues, it is easy for people to be attracted by the viewpoints and topics they accept and fall into them unconsciously. Therefore, in today’s increasingly popular social media, college students may not obtain different opinions due to the automatic recommendation algorithm of social media, and the sources of information may become homogenized and monolithic (20, 42). Some scholars consider this phenomenon to be an “information echo chamber” phenomenon, where social media may limit the exposure of different viewpoints and facilitate the formation of like-minded groups of users to build and strengthen a standard narrative, known as “echo chambers” (51). Psychological intervention should consider this phenomenon in college students who use social media as the primary information source.

### The effect of positive media content on PTSD symptoms

In some cases, after hearing or seeing some information about the catastrophe， we may experience strong empathy, evoke related traumatic experiences in the past, and generate emotions about the suffering of trauma or falling into a post-traumatic state. A study found that exposure to disaster-related social media content, including messages related to drowning persons and the emotional responses of residents, was associated with PTSD (21). On the other hand, viewing images of the storm and heroic behavior was significantly associated with lower odds of PTSD. This result puts forward higher requirements for the professionalism of the media, which should minimize the dissemination of sad messages, reduce excessive trauma, and avoid vicarious trauma.

This study used ordinal logistic regression analysis; regardless of whether gender, region of residence, and “the time point of beginning to pay attention to COVID-19” had effects on the results, both “adequate protective equipment” and “public awareness of protection” reduced PTSD symptoms in group 2. The risk of occurrence of the group 3 symptoms was also reduced by “public awareness of protection.” This result reminds us that the media’s dissemination of optimistic information can be a positive factor for students to maintain optimism and has a psychological support role. In addition, the overall proportion of students who regard being with their families as an optimistic factor is the least, which is somewhat unexpected. It may be related to the long-term quarantine at home and the outbreak of more potential intra-family conflicts.

Interestingly, in group 3, ‘other’ factors increased the risk of PTSD symptoms, a consistent result in both models. This is a question worthy of further exploration. Under the limitations of this study, some factors may not have been taken into account but potentially influence the risk of PTSD symptoms.

## Conclusion

Compared with traditional media, although the environment for individuals to obtain information has gotten rid of the closed state, the high technicalities of information have created new problems and worries. In the current environment of information overload, when college students are imprisoned and enclosed in their own “information echo chamber,” it is easy to make people blindly confident or narrow-minded, exclude others, and think that their prejudice is the truth (51).

For college students in the crisis of the epidemic, the first thing to do is to avoid sudden, excessive, and long-term negative information involvement. At the same time, it is necessary to enrich information exchange channels and moderately reduce dependence on a single media. Obtaining information by selecting popular, moderate and objective, and screened media is conducive to breaking the barriers of narrow information. Ultimately, it can help reduce the risk of panic, anxiety, and PDST symptoms to a certain extent.

The media should minimize the dissemination of pessimistic, negative, or exaggerated information, reduce excessive trauma, and avoid vicarious trauma in the audience or readers.

Schools and mental health organizations must pay special attention to the unique needs of college students with PTSD symptoms. Efficient screening and evaluation mechanisms are necessary, followed by timely help and referral to medical institutions.

## Data availability statement

The original contributions presented in the study are included in the article/Supplementary material, further inquiries can be directed to the corresponding author.

## Ethics statement

The studies involving human participants were reviewed and approved by the Ethical Committee of Research with Shanghai Mental Health Center (2020-33). Written informed consent for participation was not required for this study by the national legislation and the institutional requirements.

## Author contributions

X-LZ conducted the online questionnaires tests. W-BY analyzed the data. W-BY and ZW revised and finalized the manuscript. All authors read and approved the final manuscript.

## Funding

This research was supported by the Fudan University Affiliated Huashan Hospital Institutional Startup Fund (2020QD122), 2020YJZX0211 Research Project on Aging and Maternal and Child Health in Shanghai, EK2022ZX06 Construction Projects of Six Clinical Centers -- Child Development And Rehabilitation Clinical Center, 2020 Jiangsu University Philosophy and Social Science Research Project (2020SJB0166), 2020 Jiangsu Province Social Science Application Research Excellent Project (20SYC-139), and 2020 Shanghai Mental Health Center Science and Education Research Project (2020-YJG02).

## Conflict of interest

The authors declare that the research was conducted in the absence of any commercial or financial relationships that could be construed as a potential conflict of interest.

## Publisher’s note

All claims expressed in this article are solely those of the authors and do not necessarily represent those of their affiliated organizations, or those of the publisher, the editors and the reviewers. Any product that may be evaluated in this article, or claim that may be made by its manufacturer, is not guaranteed or endorsed by the publisher.
