# Erratum: Effects of media exposure on PTSD symptoms in college students during the COVID-19 outbreak

**DOI:** 10.3389/fpubh.2023.1346310

**Published:** 2023-12-13

**Authors:** Frontiers Production Office

**Affiliations:** Frontiers Media SA, Lausanne, Switzerland

**Keywords:** media content, mental health, COVID-19, post-traumatic stress disorder, college students, media exposure

Due to a production error, there was an error in the author list. Instead of “Wen-Bo Yu^3^” it should be “Wen-Bo Yu^4^”.

The publisher apologizes for this mistake. The original article has been updated.

