# Supplementary material for: Effects of media exposure on PTSD symptoms in college students during the COVID-19 outbreak
Source: Front Public Health. 2023 May 9;11:1050759. doi: 10.3389/fpubh.2023.1050759 (PMC10203595; doi:10.3389/fpubh.2023.1050759)
Supplement: Supplementary file 1 [file Data_Sheet_1.pdf]

Mental Health Questionnaire for Students During Covid-19 Epidemic

Dear students, due to the recent outbreak of Covid-19, we are conducting this survey on students' mental health during the outbreak. The content of the questionnaire will be kept strictly confidential. Thank you for your participation!

1. Your gender
○Male
○Female

2. Your age
○Under 18 years old
○18 years old
○19 years old
○20 years old and above

3. Your region of residence: (optional)

4. Are you a class president or a class cabinet?
○Yes, I am a Class president.
○Yes, I am a Student Union leader.
○Yes, I am a committee leader.
○No.

5. Are you knowing Covid-19?
○A lot
○A little
○None

6. When did you begin to pay attention to Covid-19?
○At the end of December 2019, the Wuhan Health Commission issued a notification for the first time that 27 people were infected with viral pneumonia.
○On January 11, 2020, the Wuhan Health Commission officially reported the first death case.
○On 19 January and 20 January 2020, cases occurred in Shenzhen and Beijing.
○On January 20, 2020, Zhong Nanshan affirmed that the new coronavirus pneumonia was human-to-human transmission and that medical staff was infected.
○On January 23, 2020, Wuhan announced a “lockdown”.
○Other or never cared

7. What are your main ways to know the latest developments of the epidemic? [multiple choice]
○Television news
○Newspaper
○Weibo, WeChat, and other social media

○Quora or Zhihu etc.
○Others

8. What information do you most want to know in your home quarantine? [multiple choice]
○Medical knowledge about effective prevention and protection of COVID-19
○Potential risk of COVID-19 in the area of oneself, family, relatives, and friends
○Efforts of doctors, nurses, NHC(National Health Commission) officials, and the police to save lives
○Operation of government to resist the COVID-19 pandemic
○Measures and experience of other countries or areas to resist the COVID-19 pandemic
○Information and statistical data on infection and spread of the COVID-19 pandemic
○Research progress of COVID-19 by professional and scientific institutions
○Analysis and interpretation of COVID-19 by experts, scholars, and professionals
○Do not care about COVID-19

9. The reasons that keep you optimistic during the Covid-19 epidemic are: [multiple choice]
○The shock of China's construction speed
○The increasing number of cured patients
○Sufficient protective appliances
○Public awareness of protection
○National unity can control the COVID-19 pandemic
○Have more time with families
○Others

10. During the Covid-19 epidemic, have you ever had any of the following? [Multiple choice]
○Efforts to avoid activities, places, and people that arouse recollections of Covid-19
○Diminished interest in activities
○Feeling of detachment from others
○Restricted affect
○Sense of foreshortened future
○Sleep problems
○Exaggerated startle response

11. How many days have you been quarantined?
○1-3 days
○4-7 days
○8-15 days
○16-30 days
○One month and above

12. How many masks have been used in your family during quarantine?
○10 or less
○10-20
○20 or more

13. What is your attitude towards online courses?
○Like
○Just to listen
○A task must be completed
○Do not like

14. Your willingness to go back to school:
○Be willing to
○Whatever
○ Do not want to return to school
